# National observational study to evaluate the “clean*your*hands” campaign (NOSEC): a questionnaire based study of national implementation

**DOI:** 10.1186/s13756-015-0077-0

**Published:** 2015-11-23

**Authors:** Christopher Fuller, Joanne Savage, Barry Cookson, Andrew Hayward, Ben Cooper, Georgia Duckworth, Susan Michie, Annette Jeanes, Louise Teare, Andre Charlett, Sheldon Paul Stone

**Affiliations:** University College London, Sheldon Stone, Royal Free Hospital, NW3 2PF. 0207 794 0500, London, UK; Formerly Health Protection Agency, London, UK; Centre for Tropical Medicine and Global Health, Nuffield Department of Clinical Medicine, University of Oxford, Oxford, UK; University College London Hospitals, London, UK; Mid-Essex NHS Trust, Chelmsford, Essex UK; Public Health England, London, UK

**Keywords:** Hand-hygiene, Implementation, Cleanyourhands campaign

## Abstract

**Introduction:**

The number of national hand-hygiene campaigns has increased recently, following the World Health Organisation’s (WHO) “Save Lives: clean your hands” initiative (2009), which offers hospitals a multi-component hand-hygiene intervention. The number of campaigns to be evaluated remains small. Most evaluations focus on consumption of alcohol hand rub (AHR). We are not aware of any evaluation reporting implementation of all campaign components. In a previously published report, we evaluated the effects of the English and Welsh cleanyourhands campaign (2004–8) on procurement of AHR and soap, and on selected healthcare associated infections. We now report on the implementation of each individual campaign component: provision of bedside AHR, ward posters, patient empowerment materials, audit and feedback, and guidance to secure institutional engagement.

**Method:**

Setting: all 189 acute National Health Service (NHS) hospitals in England and Wales (December 2005–June 2008). Six postal questionnaires (five voluntary, one mandatory) were distributed to infection control teams six-monthly from 6 to 36 months post roll-out. Selection and attrition bias were measured.

**Results:**

Response rates fell from 134 (71 %) at 6 months to 82 (44 %) at 30 months, rising to 167 (90 %) for the final mandatory one (36 months). There was no evidence of attrition or selection bias. Hospitals reported widespread early implementation of bedside AHR and posters and a gradual rise in audit. At 36 months, 90 % of respondents reported the campaign to be a top hospital priority, with implementation of AHR, posters and audit reported by 96 %, 97 % and 91 % respectively. Patient empowerment was less successful.

**Conclusions:**

The study suggests that all campaign components, apart from patient empowerment, were widely implemented and sustained. It supports previous work suggesting that adequate piloting, strong governmental support, refreshment of campaigns, and sufficient time to engage institutions help secure sustained implementation of a campaign’s key components. The results should encourage countries wishing to launch coordinated national campaigns for hospitals to participate in the WHO’s “Save Lives” initiative, which offers hospitals a similar multi-component intervention.

## Background

National hand-hygiene campaigns have increased in number recently [1, 2], following the First Global Patient Safety Challenge (2005) [3] and the World Health Organisation’s “Save Lives: clean your hands” initiative (2009) [4] which offers participating hospitals or countries a multi-component hand-hygiene intervention tool. The number of campaigns that have been evaluated remains small and mainly restricted to monitoring procurement of AHR [1]. We are not aware of any national campaign which has evaluated short or long term implementation of all campaign components. Such an evaluation would be helpful to other countries planning similar campaigns.

The English and Welsh “clean*your*hands campaign” [5, 6] was rolled out to all 189 acute NHS hospitals between 1^st^ December 2004 and 30^th^ June 2005 as a high profile national initiative reflecting public and political concern over health-care associated infections and poor hand-hygiene compliance [7]. The main components of the campaign, which was continued until December 2010, were: provision of AHR at each bedside, distribution of posters to each ward reminding health-care workers to clean their hands, audit and feedback of hand-hygiene compliance, provision of materials empowering patients to remind healthcare workers to clean their hands, and detailed guidance to help infection control teams secure institutional engagement.

The National Observational Study to Evaluate the clean*your*hands Campaign (NOSEC study) was a four year ecological study (1^st^ October 2004-30^th^ September 2008) in which we evaluated the effects of the English and Welsh cleanyourhands campaign on procurement of AHR and soap, and on selected healthcare associated infections [7, 8]. This publication however, reports on the implementation of the individual campaign components in acute NHS hospitals, at 6–36 months after roll out of the campaign, using a voluntary self-report questionnaire.

## Methods

### Study design

Repeated cross-sectional voluntary questionnaire-based survey.

### Participants

All 189 English and Welsh NHS acute hospital trusts. Data were collected at the acute trust level, a trust being the administrative unit of a small number of acute hospitals; these are described as “hospitals” throughout the report. The campaign was rolled out to all hospitals by the end of June 2005.

### Timing and distribution of questionnaire

Postal questionnaires were sent to infection control teams in December 2005 (NOSEC 1), June 2006 (NOSEC 2) December 2006 (NOSEC 3) June 2007 (NOSEC 4), December 2007 (NOSEC 5) and June 2008 (NOSEC 6). This covered the period from 6 to 36 months post completion of roll out of the campaign.

In order to maximise response rates the questionnaire was piloted in 8 acute hospitals. The National Patient Safety Agency wrote to chief executives asking that they facilitate compliance with the study ahead of the first and fourth questionnaires. Hospitals were given two months to respond to questionnaires after which they received two postal reminders. The National Audit Office, which was conducting the third mandatory survey of infection control practice in the autumn of 2008 [9], included the NOSEC questions in their own survey soon after the final questionnaire was sent out.

#### Questionnaire

Respondents were asked to respond to the following questions:

1. To what extent do you agree with the statement: “*The actions of hospital management show that the cleanyourhands campaign is a top priority in this trust*”?
2. What proportion of inpatient wards have alcohol hand rub at each bedside?
3. What proportion of inpatient wards use the cleanyourhands campaign posters?
4. To what extent do you agree with the statement *“The cleanyourhands patient empowerment materials are reaching patients on wards”?*
5. To what extent do you agree with the statement: “*The cleanyourhands campaign has encouraged patients in this hospital to ask staff to clean their hands*”?
6. In what proportion of inpatient wards has hand-hygiene compliance been directly monitored by a member of the infection control team or by a member of ward staff in the past six months?

Response options for questions 1, 3 and 4 were “strongly agree”, “agree”, “neither agree nor disagree”, “disagree” or “strongly disagree”. Potential responses to questions 2 and 5 were “none” “1–25 %”, “26–50 %”, “51–75 %”, “76–99 %”, and “100 %”.

Two extra questions were included in the National Audit Office questionnaire:

1. If you have answered less than 100 % regarding the percentage of wards with bedside AHR, in which wards is AHR unavailable at the bedside/end of bed etc.”
2. If your trust has a rolling programme of hand hygiene audits how often are they done?

Response options for the first question were “Psychiatric”, “Paediatric”, “Hepatic” and “Other”. For the second question, response options were “Weekly”, Monthly”, “Quarterly”,”Annually”, “Other”, “No rolling programme of audit”.

### Analysis

The proportion of hospitals responding to each NOSEC questionnaire was recorded, and the responses to questions on campaign implementation were expressed as a percentage of the total number of responses.

The results were assessed for attrition bias, as response rates fell, by comparing responses from consistent responders (hospitals who responded to all questionnaires) with those from inconsistent responders, using Pearson’s Chi Squared (χ^2^) and Fisher’s Exact tests for each of the first five voluntary questionnaires. The same calculations were done to compare the questionnaire returns of those who responded to the sixth voluntary questionnaire with those that responded to the mandatory National Audit Office questions.

Selection bias with respect to hospital type, using the Health Protection Agency classification of acute, teaching or specialist [7], was examined by comparing the proportions of acute, teaching and specialist hospitals responding to each questionnaire for significance (Z scores).

In order to identify potential selection bias whereby responders might also be those using more hand hygiene consumables and therefore more compliant with the campaign, the amount of AHR and soap procured (mls/patient-bed day) [7] was compared in responding and non responding hospitals for each questionnaire.

### Ethics

The study was ethically approved by Multicentre Research Ethics Committee (reference number 04MRE/10/66 Scotland). Data was anonymised and confidential.

## Results

### Response rates (Table 1)

**Table 1:** Percentage of respondents to questionnaires 1–6 reporting agreement or strong agreement with statements or reporting implementation in >75 % of wards

	NOSEC 1. 6-months post roll out	NOSEC 2. 12-months post roll out	NOSEC 3. 18 months post roll out	NOSEC 4. 24 months post roll out	NOSEC 5. 30 months post roll out	NOSEC 6. 36 months post roll out
Management’s actions show that CYHC is a top priority in Trust^a^	78 %	71 %	75 %	78 %	74 %	90 %
AHR near-patient in >75 % wards	94 %	88 %	85 %	83 %	86 %	96 %
Posters on >75 % wards	88 %	79 %	79 %	74 %	79 %	97 %
Patient empowerment materials reaching patients on wards^a^	68 %	48 %	41 %	38 %	65 %	65 %
Materials are changing patient’s behaviour^a^	46 %	49 %	46 %	34 %	41 %	35 %
Audit &feedback in last 6 months on >75 % wards	47 %	51 %	53 %	64 %	75 %	91 %
Total number (%) of responses^a^	134/189 (71 %)	126/189 (67 %)	108/187 (58 %)	99/187 (53 %)	82/185 (44 %)	167/185 (90 %)

^a^Proportions reporting agreement or strong agreement with the statement

Response rates to the voluntary questionnaires (NOSEC 1–5) were initially high but declined from 71 % (NOSEC 1) to 44 % (NOSEC 5). This rose to 90 % for the final questionnaire (NOSEC 6), 90 respondents to the National Audit Office survey and 77 respondents to the voluntary version. This gave a national “snap shot” of campaign implementation at three or more years post roll out. Fifty-three (28 %) hospitals responded to all six questionnaires.

### Campaign implementation (Table 1)

Throughout the study, the majority of infection control teams agreed or strongly agreed with the statement “*The actions of hospital management show that the cleanyourhands campaign is a top priority in this trust* and reported. Widespread implementation of bedside AHR and ward display of posters was also reported, with a steady rise in audit of hand-hygiene compliance over time. At three years or more post-roll out (NOSEC 6), the campaign still appeared to be a top priority, with near universal implementation of bedside AHR, ward posters, and audit of hand-hygiene compliance. Patient empowerment was the least successfully implemented component and most respondents did not think it had altered patients’ behaviour.

Bedside AHR was reported to be present in 100 % of wards in 22 % (NOSEC 1), 17 % (NOSEC 2), 45 % (NOSEC 3), 46 % (NOSEC 4), 49 % (NOSEC 5) and 40 % (NOSEC 6) of responding hospitals. From responses to questions included only in the National Audit Office questionnaire [9] it appeared the wards without AHR were those where there was a perceived risk of patients ingesting it. Hospitals reported that the wards without AHR were psychiatric wards (94: 56 %] hospitals), mental health wards (19: 11 % hospitals) and hepatology wards (7: 4 % hospitals).

Audit and feedback was reported to be carried out in all wards in 23 % (NOSEC 1), 25 % (NOSEC 2), 21 % (NOSEC 3), 32 % (NOSEC 4), 49 % (NOSEC 5) and 60 % (NOSEC 6) of responding hospitals. In response to questions included only in the National Audit Office questionnaire, the frequency of audit and feedback was weekly in 77 (46 %), monthly in 60 (36 %) and quarterly in 12 (7 %) of responding hospitals.

### Assessment of attrition and selection bias

There was no evidence of attrition or response bias when the returns of consistent responders and inconsistent responders were compared, or when those of responders to the voluntary and mandatory versions of the final questionnaire were compared. The only exception to this concerned responses to one question in NOSEC 2, when non-consistent responders were significantly more likely to report having campaign posters on >75 % of wards than consistent responders (χ^2^ = 7.66, *p* = 0.006). There was no evidence of selection bias with respect to type of hospital or the amount of AHR or soap procured.

## Discussion

The principal findings of the study were that hospitals reported widespread and sustained implementation of all main components of the campaign, with the exception of patient empowerment. Institutional engagement, as reflected by infection control teams’ perception of management’s actions to support the campaign, was reported to be high, even at three years after completion of national roll out. Audit and feedback was reported to have risen thoughout the study until it was reported to be widespread on nearly all wards.

The strengths of the study included use of a mandatory final questionnaire with a high response rate to provide a national “snapshot” of institutional engagement and campaign implementation at 3 years post roll out. Other strengths were that measures were taken to maximise the voluntary questionnaire response rate (providing a short questionnaire, asking the National Patient Safety Agency to write to chief executives, issuing two reminders to late returning hospitals) and that potential attrition and selection biases were assessed. Systematic review shows that 85 % of health care worker questionnaire studies fail to take measures to maximise response rates with only 16 % formally assessing attrition or selection bias [10].

The main limitation of the study was its use of self-reported data. Although national self-report questionnaires run the risk that respondents may overestimate the quality of their service, they remain a standard tool of health service research, and their findings have been used to drive national policy in infection control and other fields [9, 11–14]. The experience of all three questionnaire-based National Audit Office reports on infection control, in particular, showed that infection control teams consistently revealed widespread deficiencies in their services [9, 11, 12] and were often highly critical of management’s attitudes towards infection control. In this context, the finding in the mandatory final questionnaire that 90 % of infection control teams considered that their hospital’s management viewed the campaign as a top priority three years after national roll-out is striking and indicates that the campaign was successful in securing institutional engagement.

The second main limitation was the falling response rate to the voluntary questionnaires. This is a well-recognised trend in questionnaire-based research over the last decade, attributed to the increased demands for information in general from healthcare workers [10]. Although all response rates to the voluntary NOSEC questionnaires were below the 75 % conventionally thought necessary to minimise bias [10], extensive analyses were undertaken to exclude bias and provide reassurance that the responses are representative of the national picture. The initial response rate of 71 % was well above the median of 50 % and at the high end of the interquartile range (37–71 %) reported in the literature for nurse-directed questionnaires [10], and remained above this for the first four questionnaires. There was no difference in reported implementation between voluntary and mandatory returns, which might suggest that making reporting mandatory for those trusts that had not already voluntarily reported did not lead them to report higher levels of implementation. Hospitals reported poor implementation of many other aspects of infection control in their responses to the same mandatory National Audit Office questionnaire [9] that included the final NOSEC questionnaire. This suggests that the rise in implementation of the campaign components was genuine. It is not clear why implementation of nearly all components was reported to be higher in the final questionnaire, although it is possible that this represents a long term and cumulative change in culture.

The final limitation was that, because of the much faster than anticipated rollout, it was not possible to collect baseline data for the poster, patient empowerment, and audit components in the period prior to the introduction of the campaign. Although it is possible that some hospitals already had local initiatives incorporating elements of the campaign, it seems implausible that these were widespread, especially given the very low levels of AHR procurement pre-campaign [7].

It is difficult to compare this study with other reports evaluating national campaigns, as many have not examined implementation of these individual campaign components [15–17]. There are data, however, from the German national campaign, which reported that over three years, availability of AHR at the bedside had risen to 100 % in intensive care units and to 91.3 % in other wards [2], and that audit was carried out in 180 of over 700 hospitals. A voluntary questionnaire distributed at three years into the campaign to 450 out of over 700 hospitals, received 211 replies (47 %), reporting active management support for the campaign in 62 % of responding hospitals, with nearly 40 % reporting no managerial support. A single hospital study from Mali evaluating a very similar hand hygiene campaign, also used a self report questionnaire which also indicated that the campaign had been associated with widespread bedside availability of AHR, poster display and audit and feedback but less successful implementation of patient empowerment [18]. We too report that the patient empowerment component was the most poorly implemented. This finding may reflect the relative absence in campaign materials of detailed guidance on how to achieve this. It is also consistent with research showing that there appear to be more many more barriers than facilitators for patient empowerment in hand hygiene, and that it was “generally perceived as one of the most challenging roles” for patients to adopt, compared to other patient empowerment roles [19]. Further research carried out for the National Patient Safety Agency on patient empowerment came to the same conclusion, recommending to the Chief Medical Officer that further work to expand this component be terminated (report available from authors).

The findings of this study are best understood alongside those of the rest of the NOSEC study [7], which showed that procurement of soap and alcohol hand rub tripled, and that the rate of change of procurement at the individual hospital level increased significantly in association with each phase of the campaign. Taken together, the studies appear to show that the campaign was effectively implemented and sustained long term.

Although analysis of the mechanisms responsible for its effective long term implementation are beyond the scope of this study, systematic review of smaller scale hand hygiene interventions has suggested that frequent refreshment of interventions may be needed [20]. Guidelines for implementing and evaluating complex interventions stress the importance of a pilot phase [21] and government support for national hand hygiene campaigns in France, Belgium and Germany was identified [2, 22] as central to their success. The piloting of the campaign in a small number of hospitals [23], the three month preparation time given to infection control teams to engage their institution, and the ongoing co-ordination and support given by a dedicated centrally funded national campaign team with two refreshments or relaunches of the campaign and strong governmental support [7], may all therefore have been factors in securing sustained implementation of the campaign’s key components.

## Conclusions

The World Health Organization’s “Save Lives” initiative [4], which has been taken up by over 170 countries, offers a multi-component intervention very similar to the English and Welsh cleanyourhands campaign. The findings of this study suggest that a national campaign can successfully influence and sustain acute hospitals’ promotion of hand-hygiene. These results should encourage countries wishing to launch coordinated national campaigns for hospitals to participate in the WHO’s “Save Lives” initiative, the implementation of whose components they could evaluate themselves through the WHO hand-hygiene intervention self-assessment tool [24].

## Abbreviations

NOSEC: National Observational Study to Evaluate the clean*your*hands Campaign
NHS: National Health Service
AHR: Alcohol hand rub

## References

[1] Latham JR, Magiorakos A-P, Monnet DL, Alleaume S, Aspevall O, Blacky A (2014). The role and utilisation of public health evaluations in Europe: a case study of national hand-hygiene campaigns. BMC Public Health.

[2] Reichardt C, Koeniger D, Bunte-Schoenberger K, van der Lindena P, Moench N, Schwabb F (2013). Three years of national hand-hygiene campaign in Germany: what are the key conclusions for clinical practice?. J Hosp Infect.

[3] WHO|Clean Care is Safer Care The first global patient safety challenge. 2005; Available: http://www.who.int/patientsafety/events/05/GPSC_Launch_ENGLISH_FINAL.pdf (Accessed 12 August 2015).

[4] World Health Organization: Guidelines on Hand Hygiene in Health Care: a Summary. Geneva: World Health Organization; 2009. http://www.who.int/gpsc/5may/tools/who_guidelines-handhygiene_summary.pdf.

[5] National Patient Safety Agency [NPSA]. Ready, Steady, Go. The full guide to implementing the cleanyourhands campaign in your Trust. London: NPSA. 2004.

[6] National Patient Safety Agency [NPSA]. Flowing with the go. The complete year two campaign maintenance handbook for cleanyourhands partner trusts. The sequel to Ready, steady, go. London: NPSA. 2006.

[7] Stone SP, Fuller F, Savage J, Cookson B, Hayward A, Cooper B, et al. Evaluation of the national Cleanyourhands campaign to reduce Staphylococcus aureus bacteraemia and Clostridium difficile infection in hospitals in England and Wales by improved hand hygiene: four year, prospective, ecological, interrupted time series study. Br Med J. 2012;344, e3005. 10.1136/bmj.e3005 PMC3343183 22556101

[8] Stone S, Fuller C and the NOSEC/FIT investigators. Report to the Patient Safety Research Programme. University of Birmingham, Patient Safety Programme. Report number PS029. 2010 http://www.birmingham.ac.uk/Documents/collegemds/haps/projects/cfhep/psrp/finalreports/PS029FinalReportStone.pdf (accessed 12 August 2015)

[9] National Audit Office [NAO]. Reducing Healthcare Associated Infections in Hospitals in England. Report by the comptroller and auditor general HC 560 session 2008–9 12 June 2009. 2009

[10] Cook JV, Dickinson HO, Eccles MP (2009). Response rates in postal surveys of healthcare professionals between 1996 and 2005: An observational study. BMC Health Serv Res.

[11] National Audit Office [NAO]. The management and control of hospital acquired infection in acute NHS trusts in England. National Audit Office. Report by the Comptroller and Auditor General - HC 230 Session 1999–2000. 2000. 10.1053/jhin.2000.0753 10706797

[12] National Audit Office [NAO]. Improving patient care by reducing the risk of hospital acquired infection. National Audit Office. Report by the Comptroller and Auditor General HC 876 Session 2003–2004: 14 July 2004. 2004.

[13] Intercollegiate Stroke Working Party. National Sentinel Stroke Audit Report 2004*.* Royal College of Physicians. 2005

[14] The Continence Working Party. The National Audit of Continence Care for Older People. Royal College of Physicians. 2006

[15] Leens E. Vous êtes en de bonnes mains: résultats de la troisième campagne nationale pour l’hygiène des mains dans les hôpitaux. Episcoop. 2010;10(1):4. [French]. Available from: http://www.iph.fgov.be/epidemio/epifr/episcoop/201001fr.pdf

[16] Leens E. Nationale campagne ter bevordering van de handhygiëne, 2008-2009. Resultaten. Brussels, Belgium: Scientific Institute of Public Health. 2009. Contract No.: D/2009/2505/63. [Flemish]. Available from: http://www.iph.fgov.be/nsih/download/HH/RAPPORT_HH20082009_nl_definitief2.pdf

[17] Roche F, Fitzpatrick, F, Increase in alcohol hand rub indicates rise in hand hygiene activity. Epi-Insight 2010: 11(5). http://ndsc.newsweaver.ie/epiinsight/kbrp32bqeif12elq5r7tbh

[18] Allegranzi B, Sax H, Bengaly L, Richet H, Minta DK, Chraiti MN, et al. World Health Organization "Point G" Project Management Committee. (2010). Successful Implementation of the World Health Organization Hand Hygiene Improvement Strategy in a Referral Hospital in Mali, Africa. Infect Control Hosp Epidemiol. 2010;31:133–41. 10.1086/649796 20017633

[19] Watt I on behalf of the Patient Involvement in Patient Safety Research Group.) A review of strategies to promote patient involvement, a study to explore patient’s views and attitudes and a pilot study to evaluate the acceptability of selected patient involvement strategies. Patient Safety Research Programme. Report number PS/034. 2009.

[20] Naikoba S, Hayward A (2001). (2001) The effectiveness of interventions aimed at increasing handwashing in healthcare workers—a systematic review. J Hosp Infect.

[21] Craig P, Dieppe P, Macintyre S, Michie S, Nazareth I, Petticrew M (2008). Developing and evaluating complex interventions: the new Medical Research Council guidance. BMJ.

[22] Magiorakos A, Leens E, Drouvot V, May-Michelangeli L, Reichardt C, Gastmeier PK, et al. Pathways to clean hands: highlights of successful hand hygiene implementation strategies in Europe. Eurosurveillance. 2010;15:19560. 20460091

[23] National Patient Safety Agency [NPSA]. Achieving our aims: evaluating the results of the pilot cleanyourhands campaign. NPSA. 2004.

[24] World Health Organization: Hand Hygiene Self-Assessment Framework 2010 http://www.who.int/gpsc/country_work/hhsa_framework_October_2010.pdf?ua=1 (accessed 12 August 2015)

